# Optimization and In Vitro Characterization of Telmisartan Loaded Sodium Alginate Beads and Its In Vivo Efficacy Investigation in Hypertensive Induced Animal Model

**DOI:** 10.3390/pharmaceutics15020709

**Published:** 2023-02-20

**Authors:** Ubaidulla Uthumansha, Kousalya Prabahar, Dilli Bhai Gajapathy, Mohamed El-Sherbiny, Nehal Elsherbiny, Mona Qushawy

**Affiliations:** 1 Department of Pharmaceutics, Crescent School of Pharmacy, B.S. Abdur Rahman Crescent Institute of Science and Technology, Chennai 600048, India; 2 Department of Pharmacy Practice, Faculty of Pharmacy, University of Tabuk, Tabuk 71491, Saudi Arabia; 3 Formulation Research & Development, Aurobindo Pharma, Hyderabad 500038, India; 4 Department of Basic Medical Sciences, College of Medicine, Almaarefa University, Riyadh 13713, Saudi Arabia; 5 Department of Anatomy and Embryology, Faculty of Medicine, Mansoura University, Mansoura 35516, Dakahlia, Egypt; 6 Department of Pharmaceutical Chemistry, Faculty of Pharmacy, University of Tabuk, Tabuk 71491, Saudi Arabia; 7 Department of Biochemistry, Faculty of Pharmacy, Mansoura University, Mansoura 35516, Dakahlia, Egypt; 8 Department of Pharmaceutics, Faculty of Pharmacy, University of Tabuk, Tabuk 71491, Saudi Arabia; 9 Department of Pharmaceutics, Faculty of Pharmacy, Sinai University, Alarish 45511, North Sinai, Egypt

**Keywords:** telmisartan (TEL), gastroretentive beads, bioavailability, central composite design, in vitro buoyancy, anti-hypertension, alginate polymer

## Abstract

Background: Antihypertensive drug telmisartan (TEL) belongs to BCS class II, which is characterized by low water solubility and, consequently, low oral bioavailability. Gastroretentive systems may overcome the problems associated with low solubility of TEL and incomplete absorption by localizing the drug release in the stomach. The purpose of this study was to prepare TEL-loaded, oil-entrapped, floating alginate beads with the intent of enhancing the oral bioavailability of TEL for the treatment of hypertension. Methods: For the formulation and optimization of seventeen formulations of TEL-loaded oil-entrapped floating alginate beads, a central composite design was utilized. The concentration of sodium alginate (X1), the concentration of cross-linker (X2), and the concentration of sesame oil (X3) served as independent variables, whereas the entrapment efficiency (Y1), in vitro buoyancy (Y2), and drug release Q6h (Y3) served as dependent variables. Using the emulsion gelation method and calcium chloride as the cross-linking agent, different formulations of TEL alginate beads were produced. All formulations were evaluated for their entrapment efficiency percentage, in vitro buoyancy, and in vitro drug release. The optimal formulation of TEL alginate beads was prepared with and without oil and evaluated for entrapment efficiency percentage, in vitro buoyancy, swelling ratio, average size, and in vitro drug release. Using scanning electron microscopes, the surface morphology was determined. Using IR spectroscopy, the compatibility between the ingredients was determined. In vivo evaluation of the optimized formulation in comparison to the free TEL was done in hypertension-induced rats, and the systolic blood pressure and all pharmacokinetic parameters were measured. Results: The prepared beads exhibited a high entrapment efficiency percentage, in vitro buoyancy, and prolonged drug release. TEL was compatible with other ingredients, as approved by IR spectroscopy. The prepared TEL beads were spherical, as shown by the SEM. The relative bioavailability of TEL-loaded oil-entrapped beads was 222.52%, which was higher than that of the pure TEL suspension. The prepared TEL beads formulation exhibited a higher antihypertensive effect for a prolonged time compared to pure TEL suspension. Conclusions: It can be concluded that this innovative delivery method of TEL-loaded oil-entrapped beads is a promising tool for enhancing drug solubility and, thus, oral bioavailability and therapeutic efficacy, resulting in enhanced patient compliance. Furthermore, the in vivo study confirmed the formulation’s extended anti-hypertensive activity in animal models.

## 1. Introduction

Oral medication is the most common form of drug administration due to benefits such as ease, patient preference, cost-effectiveness, and simplicity of mass production [1]. Despite these benefits, the development of oral formulations presents a number of obstacles [2]. These are primarily attributable to the physicochemical properties of drugs, such as their poor water solubility and membrane permeability [3]. Several techniques were used to enhance the drug solubility after oral administration and, hence, enhance their bioavailability [4]. These strategies include amorphous solid dispersions using synthetic polymers [5], the co-crystal technique [6], electrospinning [7], and floating beads [8]. Low-density floating beads have sufficient buoyancy to float over gastric contents and remain in the stomach for an extended period of time. As a result, the drugs are slowly released from the system at the desired rate, resulting in increased gastric retention and minimal fluctuations in plasma drug concentration [9].

Telmisartan (TEL) is an oral anti-hypertensive agent that belongs to class II of the biopharmaceutical classification system (BCS) and is characterized by a low water solubility [10]. TEL’s solubility in aqueous solutions is highly pH-dependent, with maximum solubility occurring at high and low pH values. Its solubility in water is 0.078 mg/mL [11]. It has a limited and variable oral bioavailability of 42–58% due to its poor water solubility after oral administration [12]. The poor solubility of TEL leads to poor dissolution; hence, it shows variations in bioavailability [13]. The drug has a pH-dependent solubility and it is insoluble at higher pH values from three to nine [14]. Formulating TEL as gastroretentive beads aids in solubilizing the drug in acidic environments by prolonging the gastric retention time, thereby improving the bioavailability of the drug [15]. Gastroretentive drug delivery system is a new drug delivery technology that is advantageous because of its ability to maintain prolonged stomach retention, hence enhancing drug stomach residence time and drug bioavailability. To increase stomach residence time, different technological methods, including swelling and expansion, mucoadhesive, high density, ion exchange, magnetic, and floating drug delivery systems, are employed [16]. Floating beads, low-density drug delivery systems, can float in the gastric fluids, allow the solubility of the drug in the acidic medium of the stomach, and hence improve its bioavailability [17].

The solubility of TEL can be improved by various techniques, such as surface solid dispersions, co-crystals, etc. [18], whereas formulating it as oil-entrapped beads not only improves solubility but also aids in achieving the sustained activity of the drug [19,20,21]. Sodium alginate is used as a polymer to localise and retain the drug in acidic environments; the polymer is selected due to its biocompatibility [22,23], biodegradability, mucoadhesive property [24], and nontoxicity [25,26,27]. 

In the process of gelation with divalent ions such as calcium, hydrocolloid molecules such as alginate go through an instantaneous hydration process, which results in the formation of a hydrocolloidal layer with a high viscosity [28]. This results in the formation of a diffusion barrier, which reduces the movement of small molecules, such as drugs. Within a polymer matrix that regulates the drug’s release rate, the active ingredient is evenly spread. Following the contact with the dissolution medium, drug release is shown to be controlled by diffusion occurring within the insoluble matrix [29].

Alginate carboxyl groups are protonated in acidic environments and loosen the matrix structure. This is overcome by the addition of oils, which increases the complex between drug and alginate chains prolonging drug diffusion from the polymer matrix [30]. The inclusion of oil also imparts buoyancy to the beads, which leads to their enhanced gastric retention [31]. Sesame oil was utilized as a dispersed phase to generate a uniform emulsion to create multiple tiny chambers in the alginate matrix for better buoyancy [32]. Additionally, the incorporation of oil makes it easier to generate homogenous beads with better diffusion and swelling rates, which leads to an improvement in the overall performance of the process [33]. 

The primary purpose of this research was to construct novel TEL-loaded oil-entrapped floating alginate beads with the intention of increasing the bioavailability of the medication when taken orally. In order to improve both solubility and absorption, the use of the floating beads helped to achieve a prolonged release of the medicine in the acidic environment of the stomach. A central composite design was used in conjunction with a number of different sodium alginate concentrations, cross-linker concentrations, and oil concentrations in order to achieve the goal of producing an optimum formulation of TEL-loaded oil-entrapped floating alginate beads. The TEL-loaded oil-entrapped floating alginate beads were examined to determine their drug entrapment efficiency %, in vitro buoyancy, in vitro drug release, and in vitro drug release kinetics. The FT-IR was used for the compatibility investigations between the drug and the other ingredients. A scanning electron microscope (SEM) was used to determine the surface morphology of the prepared beads. The effectiveness of the optimized formulation was examined in hypertension induced rats in comparison with the free TEL suspension. 

## 2. Materials and Methods

### 2.1. Materials

Telmisartan powder was received as a gift sample from Fourrts India Pharmaceuticals Ltd. (India). Sodium alginate was purchased from Sigma–Aldrich, (St. Louis, MO, USA) with heavy metals (max 0.003%), sesame oil (heavy metals, %: 0.001 max), and Tween 80 (viscosity, 400–620 mPa·s) from Fourrts India Pharmaceuticals Ltd. (TN, India). Calcium chloride was purchased from Dae Jung Chemicals and Metals (SEL, Republic of Korea). All other chemicals were of analytical grade. 

### 2.2. Experimental Part

#### 2.2.1. Design for TEL-Loaded Oil-Entrapped Floating Alginate Beads Based on the Central Composite Design

Response surface methodology was used to design and develop optimal oil-entrapped beads as a potential gastro-retentive delivery system. The computational-design strategy was created using rotatable central composite design with an axial value of 1.682. Design-Expert software version 11 (Stat-Ease, Minneapolis, MN, USA) (https://www.statease.com/docs/v11/) was used for designing the formulations. Three formulation factors, concentrations of sodium alginate (X1), cross-linker concentration (X2), and the concentration of sesame oil (X3), were used. The effects of formulation factors on the responses, entrapment efficiency (Y1), in vitro buoyancy (Y2), and drug release Q6h (Y3) were studied to select the optimal formulation. See Table 1.

#### 2.2.2. Preparation of TEL-Loaded Oil-Entrapped Floating Alginate Beads

The TEL-loaded oil-entrapped floating alginate beads were formulated by emulsion-gelation method using calcium chloride (CaCl_2_) as a cross-linking agent [21]. In this method, a sodium alginate solution with various concentrations was prepared by dissolving an accurate amount of sodium alginate in distilled water at room temperature using a magnetic stirrer (Remi Motor, Mumbai, India). An accurate amount of TEL (100 mg) was dispersed in sesame oil and mixed with the aqueous solution of sodium alginate in the presence of Tween 80 (0.5%) as an emulsifier by high-shear mixing for 20 min at 2000 rpm using a magnetic stirrer to form an O/W emulsion. The emulsion mixture was extruded into a calcium-chloride solution under mild stirring using a syringe G 21 needle [21]. The gel beads were allowed to stand in the solution for 15 min to complete the curing reaction and to produce rigid beads. The wet gel beads were collected by decantation, washed thrice with 100 mL distilled water, and dried in the incubator at 40 °C for 24 h. The experimental conditions including stirring speed, the distance between the syringe and cross-linking solution level, the number of drops extruded at each minute, and the temperature were kept constant for all formulations. Table 2 shows the composition of the designed formulations of TEL-loaded oil-entrapped floating alginate beads based on central composite design.

#### 2.2.3. Characterization of TEL-Loaded Oil-Entrapped Floating Alginate Beads

##### Determination of the Entrapment Efficiency Percentage (Y1) of TEL-Loaded Oil-Entrapped Floating Alginate Beads

To determine the entrapment efficiency percentage, a 100-milligram sample of the prepared beads was crushed in a mortar. The crushed substance was dissolved in 75 mL of 0.1 N HCl, after which the volume was raised to 100 mL [34]. This mixture was filtered and subsequent dilutions were made before evaluation using a UV/visible spectrophotometer (Shimadzu, Kyoto, Japan) at λ_max_ 296 nm against 0.1 N HCl as a blank. The entrapment efficiency percentage can be calculated using Equation (1):

(1) $$\mathrm{EE}\%\;=\left(\frac{\mathrm{Actual}\;\mathrm{drug}\;\mathrm{content}}{\mathrm{Theoretical}\;\mathrm{drug}\;\mathrm{content}}\right)\times 100\;$$

##### In Vitro Buoyancy Study of TEL-Loaded Oil-Entrapped Floating Alginate Beads at pH 1.2 (Y2)

The TEL-loaded oil-entrapped floating alginate beads (50 beads of each formulation) were submerged in 900 mL of simulated gastric fluid (pH 1.2) in USP type II equipment (Electrolab, Mumbai, India) at a rotational speed of 50 rpm while the temperature was kept at 37 ± 5 °C. The simulated gastric fluid was prepared by dissolving NaCl (3 g) in 1450 (mL) of deionized water and then adjusting the pH to 1.2 ± 0.1 with diluted HCl [35]. Beads were observed visually as was the total floating time; the time taken by the prepared beads to remain on the surface of the medium was evaluated using a stopwatch [36]. Where the time taken by the beads to emerge on the surface of the medium is referred to as floating lag time, and the time taken by the floating beads to float constantly on the surface of the media is referred to as the total floating time. 

##### In Vitro Drug-Release Study of TEL-Loaded Oil-Entrapped Floating Alginate Beads (Y3)

The dissolution rate of the prepared TEL beads was studied using USP basket dissolution apparatus I. An accurate weight of beads equivalent to 40 mg of TEL was placed in the cup containing 900 mL of buffer pH 1.2 as a dissolution medium. The dissolution medium was kept at 37 ± 0.5 °C and stirred at 50 rpm. At predetermined time intervals (1, 2, 3, 4, 5, 6, 7, 8, 9, 10, 11, and 12 h), 5 mL was withdrawn from the cup and replaced with a fresh dissolution medium to keep the sink condition [37]. The samples were diluted and analyzed spectrophotometrically for the amount of drug released at 296 nm. The experiment was conducted in triplicates and the mean ± SD was calculated. 

#### 2.2.4. Selection of the Optimized Formulation of TEL-Loaded Oil-Entrapped Floating Alginate Beads

With the help of Design-Expert 11 software, a central composite design was used to find the best level of the formulation factors (X1, X2, and X3) for making the TEL-loaded oil-entrapped floating alginate beads that gave the desired response.

##### Preparation and Evaluation of the Optimized Formulation (OP1 and OP2)

The selected optimized formulation was prepared according to its composition by the emulsion-gelation method, as mentioned above. The optimum levels of formulation factors for an optimized formulation based on the central composite design were 4.56% sodium alginate, 8.72% calcium chloride, and 7.68% oil. Two optimized formulations were prepared according to the previous composition with and without the presence of oil (OP1 and OP2) to study its effect on the characterization of the beads and anti-hypertensive efficiency. The prepared OP1 and OP2 were evaluated for entrapment efficiency percentage, in vitro buoyancy, swelling ratio, average size, and in vitro drug release, as mentioned above. The in vitro release data of OP1 and OP2 were fixed into various mathematical models, such as zero-order, first-order, Higuchi, Korsemeyer–Peppas, and Hixson–Crowell to study the best-fitting mathematical model for the release profile. The Korsemeyer–Peppas Equation (2) can be expressed as:

(2) $$F\;=\left(M_{t}/M\right)={\;K}_{m}{\;t}^{n}\;$$

where F is the fraction of drug released at time t, M_t_ is the amount of drug released at time t, M is the total amount of drug in dosage form, K_m_ is the kinetic constant, n is the diffusion or release exponent, t is the time in hours, and n is estimated by linear regression of log (M_t_/M) against log t. If n = 0.45, there is a Fickian diffusion, while 0.45 < n < 0.89 suggests a non-Fickian diffusion or anomalous diffusion. Anomalous diffusion, also known as non-Fickian diffusion, is a combination of diffusion-controlled and erosion-controlled rate laws. A straight line was shown between the log-time release on the *x*-axis and the log cumulative percentage of drug release on the *y*-axis.

##### Determination of the Average Size of Optimized Formulation (OP1 and OP2)

The prepared beads (n > 100) were lined and the diameter was determined by a digital caliper (ROHS CE Digital Caliper–SH20, Shanghai, China). Measurements for each sample were performed in triplicate. The mean diameter and its standard deviations were recorded [38,39]. 

##### Determination of the Swelling Characteristics of Optimized Formulation (OP1 and OP2)

The swelling characteristics of the optimized formulation (OP1 and OP2) were investigated. Accurate weighted samples were placed in a wire basket of the USP dissolution apparatus II. The bead-filled basket was placed inside a beaker, which had 100 mL of 0.1 N hydrochloric acid with a pH of 1.2 and was heated to 37 °C [34]. The beads were periodically removed from the basket and weighed at regular predetermined intervals (1, 2, 3, 4, 5, and 6 h). The swelling ratio was calculated using Equation (3):

(3) Swelling ratio = Weight of wet beads/ Weight of dried beads

##### Determination of Surface Morphology of the Optimized Formulation using Scanning electron microscopy (SEM)

The surface morphology of the optimized formulation was analyzed by SEM. The beads were attached to a stub using double-sided adhesive tape, and gold was encrusted under a vacuum in an ion sputter with a thin layer of 3–5 nm of gold for 75 s. The images were captured randomly using SEM at an acceleration voltage of 10 kV [40].

- Infrared (IR) Spectroscopy Study

IR spectroscopy was used to investigate the probability of drug interaction with other substances in the manufactured TEL-loaded oil-entrapped beads [41]. Infrared spectroscopy was used to characterize the drug, sodium alginate, and the optimized formulation. Using a Thermo Scientific Nicolet IR 200 spectrometer, each sample was subjected to IR spectroscopy after being crushed into discs with potassium bromide (KBr). Every compressed sample was scanned between 4000 cm^−1^ to 400 cm^−1^.

### 2.3. In Vivo Study

#### 2.3.1. Experimental Animals

To evaluate the in vivo performance of the optimized TEL-loaded beads, a pharmacodynamics study was performed in an animal model at C.L Baid Metha College of Pharmacy.

All experimental procedures were approved by CPCSEA/INSTITUTIONAL ANIMAL ETHICAL COMMITTEE (IAEC). Approval number: 09/321/PO/Re/S/01/CPCSEA.

Adult Wistar albino rats (male and female) (220–250 g) were procured from the animal house of the C.L Baid Metha College of Pharmacy (Chennai, India) and kept under standard laboratory temperature (25 ± 1 °C), humidity (55 ± 5%), and lighting (12-h light/dark cycle) conditions. Four animals were housed per polypropylene cage and were allowed free access to water and a standard laboratory diet. Animals were kept for 7 days to acclimatize prior to treatment administration.

#### 2.3.2. In Vivo Pharmacokinetics Study

Animals were divided into three groups (n = 6), each comprising 3 males and 3 females. Group I received the pure drug TEL, Group II received optimized beads with oil (PO1), and Group III received optimized beads without oil (OP2). Treatments were administered in single doses of 1 mg/kg, po by oral gavage. Next, blood samples were collected at 1, 2, 3, 4, 6, 10, 12, 20, and 24 h following treatment from tail veins of rats using heparin as an anti-coagulant. Plasma samples were prepared by centrifugation for 15 min at 3500 rpm and stored at 2–10 °C. Reversed-phase high-performance liquid chromatographic (HPLC) technique was used to assess the drug content in the plasma samples [42]. The plasma sample (0.5 mL) was acidified by mixing it with 1 M HCl, followed by vortexing with 4 mL of ethyl acetate. Next, it was centrifuged at 4000 rpm for 10 min and the organic phase was separated into a clean tube and allowed to evaporate until dry at 50 °C in the presence of a gentle stream of N_2_, after which it was analyzed by HPLC [43]. 

#### 2.3.3. In Vivo Pharmacokinetic Analysis

The peak plasma concentration (C_max_), time to achieve maximum plasma concentration (T_max_), elimination rate constant K_el_, total area under the plasma concentration-time curve (AUC_0–∞_), relative bioavailability %, and the mean residence time (MRT) were all determined using plasma samples from six rats from each group. Each plasma concentration data was directly calculated into the C_max_ and T_max_. AUC_0–∞_ was calculated by adding the area from the last sample point to infinity (AUC_t–∞_) and the area from time zero to the last sampling time (AUC_0–t_) [44]. The AUC_0–t_ was calculated using the trapezoidal method, while the AUC_t–∞_ was found by dividing the last drug concentration measured in the plasma by K_el_, the total elimination rate constant [45]. The K_el_ was calculated using Equation (4), while TEL relative bioavailability (%) was calculated by Equation (5).

(4) $$K_{\mathrm{el}}=-\mathrm{Slope}\times 2.303\;$$

(5) $$\%\;\mathrm{Relative}\;\mathrm{bioavailability}=\left[\frac{{\mathrm{AUC}}_{\mathrm{test}}}{{\mathrm{AUC}}_{\mathrm{ref}}}\right]\times \left[\frac{{\mathrm{Dose}}_{\mathrm{ref}}}{{\mathrm{Dose}}_{\mathrm{test}\;}}\right]\times 100\;$$

#### 2.3.4. Efficacy of Beads against Hypertension Induced in Rats

A vet-Dop2 Doppler blood-pressure system with a sphygmomanometer and an animal blood-pressure cuff of the correct size was used to measure systolic blood pressure [46]. Initial BP values of rats were recorded and, thereafter, hypertension was induced by subcutaneous injection of Medroxy Progesterone Acetate (MPA, 10 mg/kg/week) for 2 weeks. Animals with a minimum mean BP of 167 ± 3.2 mmHg were considered hypertensive. To test the effect of treatments on hypertension, animals were assigned to five groups (n = 6). Group I was normal controls, Group II was positive controls, Group III was treated with TEL (5 mg/kg orally), Group IV received optimized beads without oil entrapment, and Group V received oil-entrapped optimized beads. The standard and the formulation were administered through oral gavage through suspension in sodium CMC for 21 days, BP was measured at 1, 2, 3, 4, 5, 6, 7, 8, 10, and 12 h following treatment. 

### 2.4. Statistical Analysis

Data were presented as mean ± standard deviation (SD). Analysis of significance among different experimental groups was performed using one-way analysis of variance (ANOVA) and followed by Tukey’s post hoc test. Significance was considered at *p*-values less than 0.05.

## 3. Results and Discussion

In this study, oil-entrapped floating alginate beads loaded with TEL were prepared using a response surface methodology and Stat-Ease Design-Expert software version 11. Using a central composite design, the important formulation variables that affected the responses, entrapment efficiency (Y1), in vitro buoyancy (Y2), and drug release Q6h (Y3) of the TEL-loaded oil-entrapped beads were investigated. The purpose of the experiment was to examine the impact of the concentrations of sodium alginate and the cross-linking agent (CaCl_2_), and the concentration of oil entrapped within the beads on the aforementioned responses. According to the central composite design, a total of 17 formulations with three central points, three factors, and three responses were developed (Table 2).

### 3.1. Study of the Effect of Formulation Factors (X1, X2, and X3) on Responses (Y1, Y2, and Y3)

**Figure 1:**
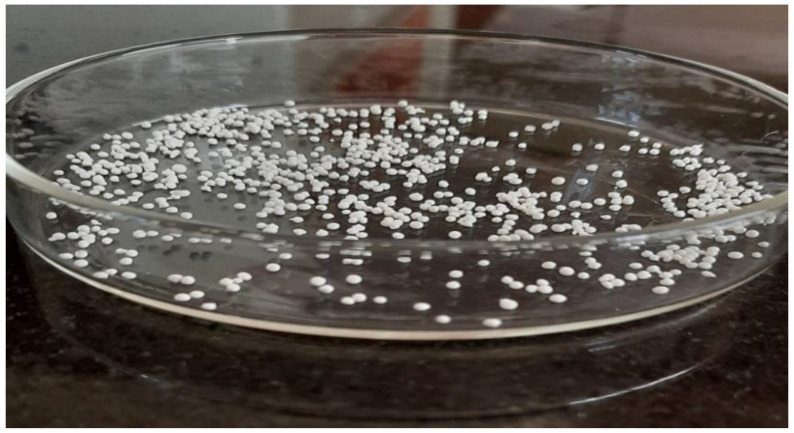
Image of the optimized TEL-loaded oil-entrapped floating alginate beads.

A total of 17 formulations were made based on the central composite design, and the results are shown in Table 2. As shown in Figure 1, the TEL-loaded oil-entrapped floating alginate beads that were made were in the shape of spheres. All of the dependent variables were found to be affected by the formulation factors. The main effect plots and three-dimensional response surfaces showed how the independent factors affected the observed responses.

#### 3.1.1. Effect of Formulation Factors (X1, X2, and X3) on Entrapment Efficiency Percentage (Y1)

**Figure 2:**
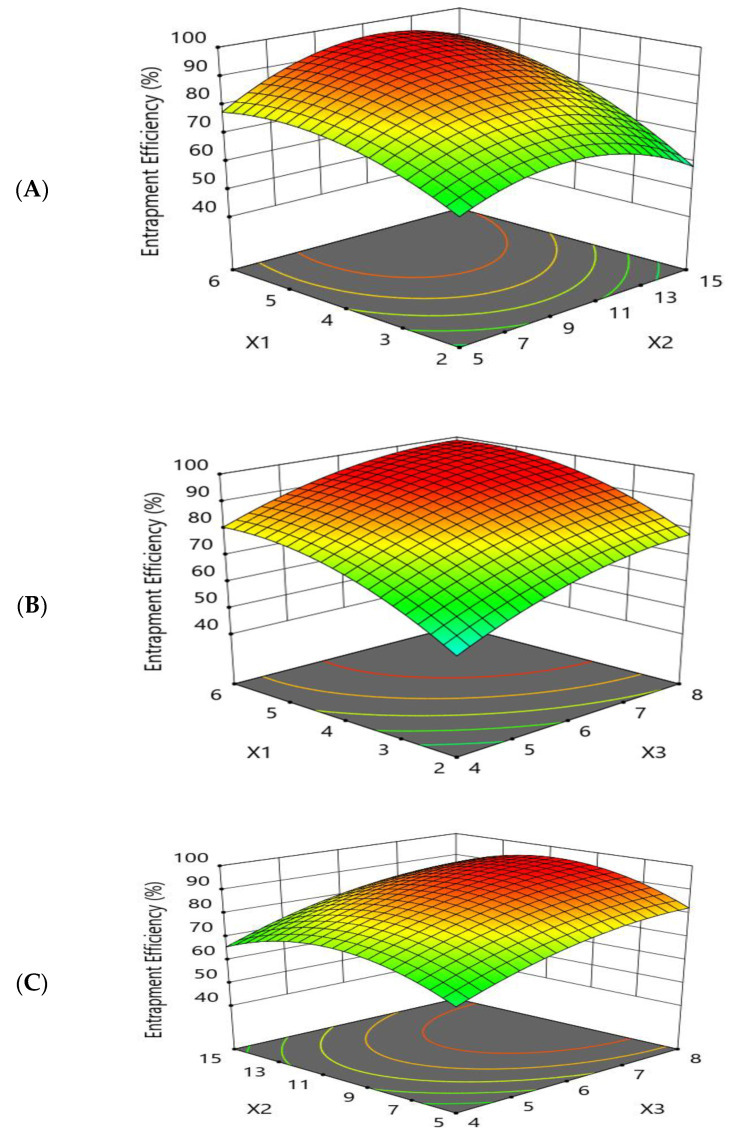
Response-surface-methodology plot for the effect of formulation factors (X1, X2, and X3) on the entrapment efficiency percentage (Y1). (**A**) Effect of sodium alginate concentration and cross-linker concentration on the entrapment efficiency percentage (Y1); (**B**) effect of sodium alginate concentration and sesame oil concentration on the entrapment efficiency percentage (Y1); and (**C**) effect of cross-linker concentration and sesame oil concentration on the entrapment efficiency percentage (Y1).

**Figure 3:**
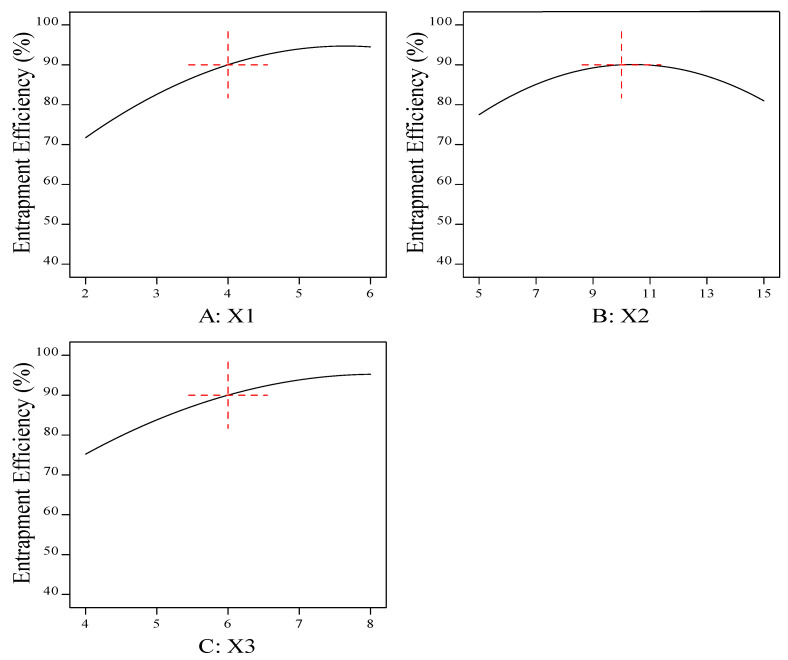
Main-effect plot for the effect of formulation factors (X1, X2, and X3) on the entrapment efficiency percentage (Y1). (**A**) Effect of sodium alginate concentration on the entrapment efficiency percentage (Y1); (**B**) effect of cross-linker concentration on the entrapment efficiency percentage (Y1); and (**C**) effect of sesame oil concentration on the entrapment efficiency percentage (Y1).

The goal of the optimization study was to maximize the entrapment efficiency percentage of the prepared beads. The effects of the independent variables on the entrapment efficiency were studied, as shown by the three-dimensional response surface and main-effect plots (Figure 2 and Figure 3).

The response-surface and main-effect plots relating to the drug-entrapment efficiency (Y1) demonstrated that increasing the polymer concentration (X1) from 2% to 6% led to an improvement in the drug-entrapment efficiency. This may be attributed to the rigid structure of the prepared beads, due to the cross-linking between the sodium alginate and the calcium chloride forming an egg-box structure of calcium alginate, which decreased the leakage of drug [47]. 

It was observed from the results that the increase in cross-linking concentration (X2) from 5% to 10% led to an increase in the entrapment efficiency percentage (Y1), while further increases in X2 up to 15% resulted in a decrease in Y1. 

Furthermore, the effect of the amount of oil (X3) on Y1 was studied. It was observed that the entrapment efficiency percentage was increased by increasing the amount of oil. These results may have been due to the diffusion of the drug into the surrounding medium during the gelation process, while the barrier activity of the entrapped oil droplets was increased by increasing the oil concentration and more of the drug was protected from diffusion, leading to an increase in the entrapment efficiency [48]. The polynomial fitting model for EE (Y1) is shown below.

$$\begin{array}{cc} \mathrm{Entrapment}\; & \mathrm{Efficiency}\;\left(\%\right)\\ & =90.0032+11.3928\times X_{1}+1.7407\times X_{2}+10.0215\times X_{3}+4.25\times X_{1}\times X_{2}\;\\ & +\left(-1\right)\times X_{1}\times X_{3}+0.25\times X_{2}\times X_{3}+\left(-6.90525\right)\times X_{1}^{2}+\left(-10.7943\right)\times X_{2}^{2}\;\\ & +-\left(4.78393\right)\times X_{3}^{2} \end{array}$$

#### 3.1.2. Effect of Formulation Factors (X1, X2, and X3) on the In Vitro Buoyancy (Y2)

The goal of the optimization was to improve the floating time of the formulation by maximizing the buoyancy of the beads as this has a significant influence on the rate of drug release. 

**Figure 4:**
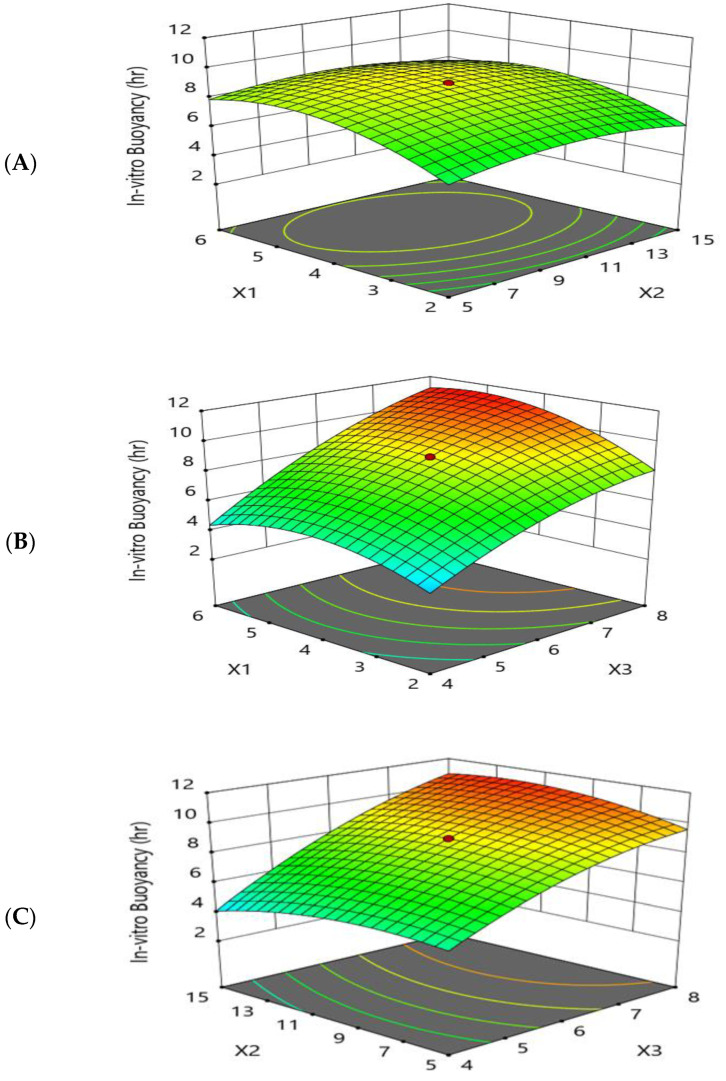
Response-surface-methodology plot for the effect of formulation factors (X1, X2, and X3) on the in vitro buoyancy (Y2). (**A**) Effect of sodium alginate concentration and cross-linker concentration on the in vitro buoyancy (Y2); (**B**) effect of sodium alginate concentration and sesame oil concentration on the in vitro buoyancy (Y2); and (**C**) effect of cross-linker concentration and sesame oil concentration on the in vitro buoyancy (Y2).

**Figure 5:**
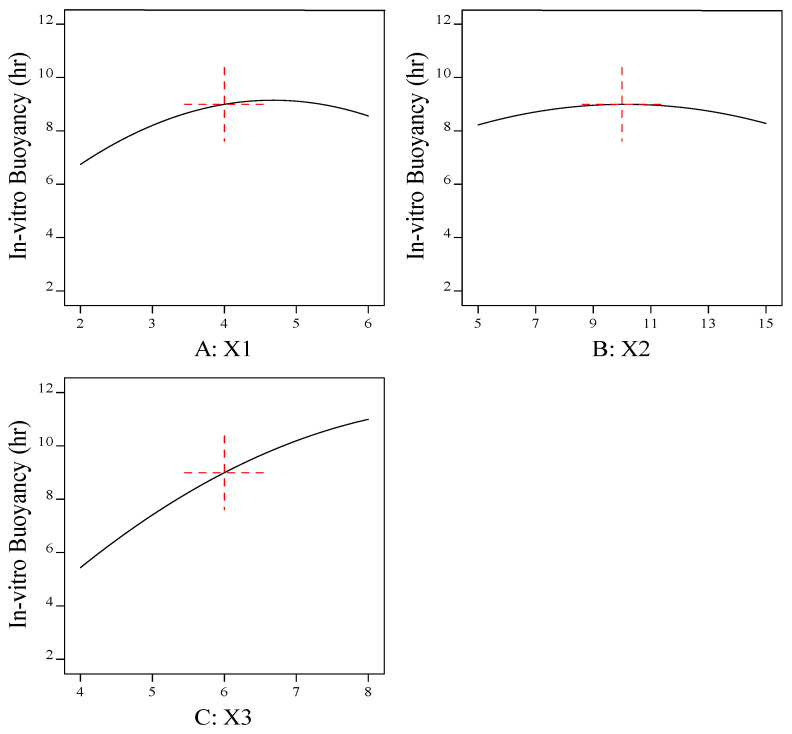
Main-effect plot for the effect of formulation factors (X1, X2, and X3) on the in vitro buoyancy (Y2). (**A**) Effect of sodium alginate concentration on the in vitro buoyancy (Y2); (**B**) effect of cross-linker concentration on the in vitro buoyancy (Y2); and (**C**) effect of sesame oil concentration on the in vitro buoyancy (Y2).

As shown by Figure 4 and Figure 5, the response-surface plots relating to the in vitro buoyancy and the main-effect plot revealed that the in vitro buoyancy time (Y2) was increased by increasing the sodium-alginate concentration (X1) and the cross-linker (X2) to moderate levels, while further increases resulted in decreases in (Y2). These results may be attributed to the higher sodium-alginate concentration and cross-linking concentration, resulting in the formation of a thicker alginate layer and increasing the weight of the beads [49]. Denser beads taper the property of buoyancy, suggesting an extended lag time and reduced floating period.

The greater the concentration of oil (X3), the greater the buoyancy and floating time (Y2). Formulations with a greater proportion of oil floated more rapidly than those with a lower proportion of oil. Another reason for the buoyancy is the swell and expansion of the matrix volume in aqueous fluids, which further reduced the density of the beads and made them float more easily. It was discovered that adding low-density oils, such as sesame oil, increased the in vitro buoyancy, allowing the beads to float for 8–12 h [50]. All formulations floated immediately as they were introduced into the simulated gastric fluid with zero or minimal lag time. The polynomial fitting model for in vitro buoyancy (Y2) is shown below.

$$\begin{array}{cc} \mathrm{In}\;\mathrm{Vitro}\;\mathrm{Buoyancy}\; & \left(h\right)\\ & =8.99569+0.909626\times {\;X}_{1}+0.0282884\times {\;X}_{2}+2.7808\times {\;X}_{3}+\left(-0.1\right)\times {\;X}_{1}\times {\;X}_{2}\;\\ & +0.625\times {\;X}_{1}\times {\;X}_{3}+0.625\times {\;X}_{2}\times {\;X}_{3}+\left(-1.34635\right)\times {\;X}_{1}^{2}+\left(-0.745306\right)\times {\;X}_{2}^{2}\;\\ & +\left(-0.780662\right)\times {\;X}_{3}^{2} \end{array}$$

#### 3.1.3. Effect of Formulation Factors (X1, X2, and X3) on the In Vitro Drug Release Q6h (Y3)

**Figure 6:**
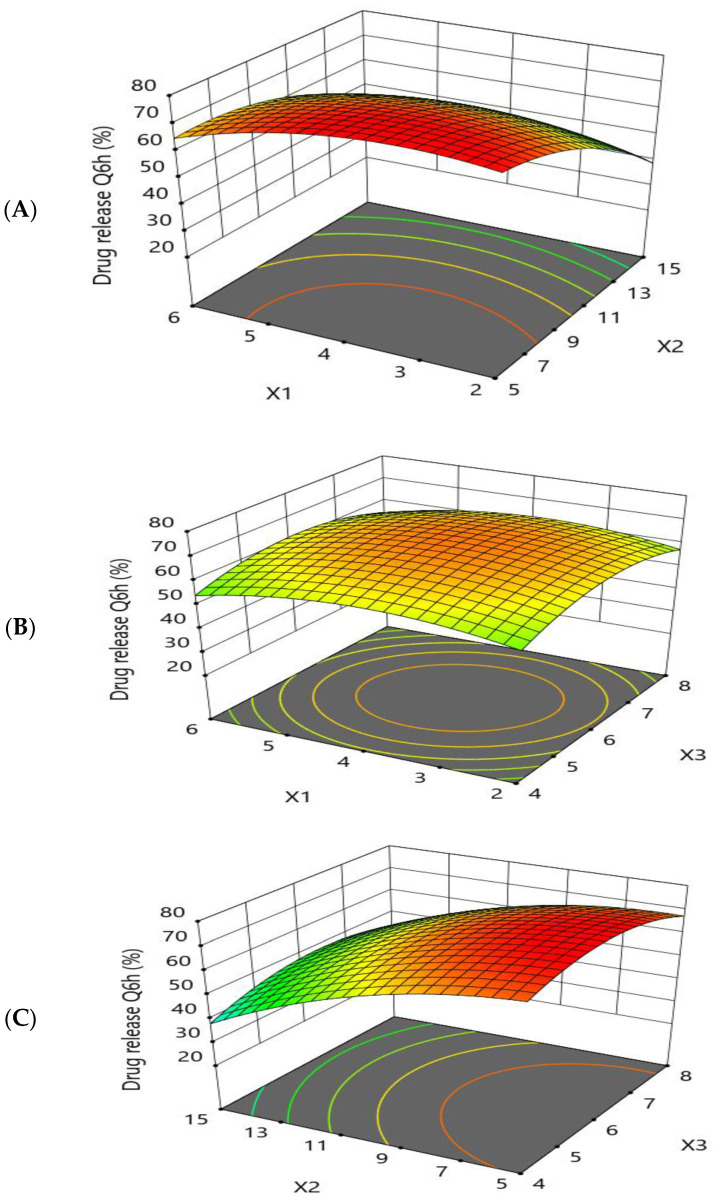
Response-surface-methodology plot for the effect of formulation factors (X1, X2, and X3) on the in vitro drug release Q6h (Y3). (**A**) Effect of sodium alginate concentration and cross-linker concentration on the in vitro drug release Q6h (Y3); (**B**) effect of sodium alginate concentration and sesame oil concentration on the in vitro drug release Q6h (Y3); and (**C**) effect of cross-linker concentration and sesame oil concentration on the in vitro drug release Q6h (Y3).

**Figure 7:**
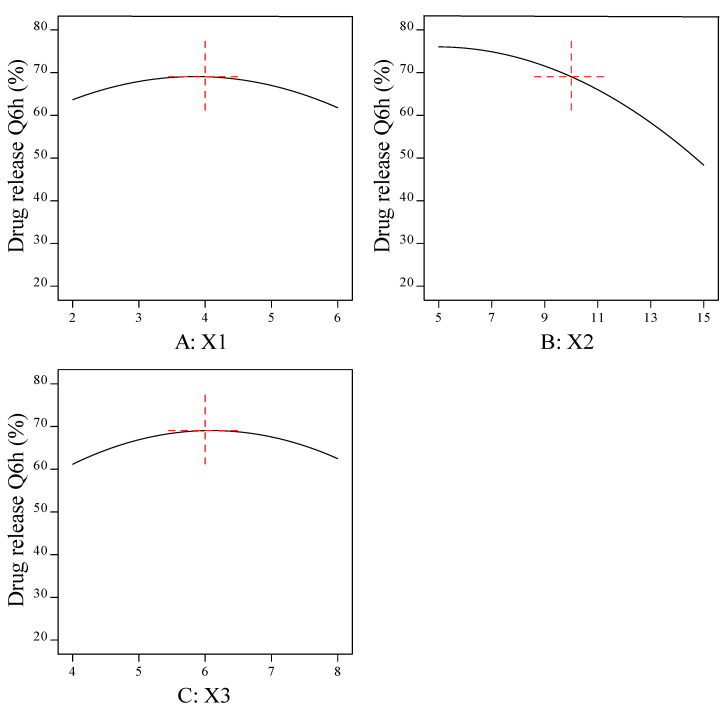
Main-effect plot for the effect of formulation factors (X1, X2, and X3) on the in vitro drug release Q6h (Y3). (**A**) Effect of sodium alginate concentration on the in vitro drug release Q6h (Y3); (**B**) effect of cross-linker concentration on the in vitro drug release Q6h (Y3); and (**C**) effect of sesame oil concentration on the in vitro drug release Q6h (Y3).

All the prepared beads exhibited prolonged drug release in the simulated gastric fluid (pH 1.2) for 12 h. The response-surface plots and main-effect plots for the effect of the formulation factors on the drug release Q6h (Y3) are depicted in Figure 6 and Figure 7, respectively. An increase in the concentration of sodium alginate (X1) up to moderate levels induced a higher drug-release rate. The reason behind this is the swelling mechanism; when introduced into an acidic medium, the alginate matrix was converted into alginic acid, which lead to a reduced gel strength and aided in the diffusion of the drug from the matrix with an accelerated rate of release [51]. Further increases in X1 resulted in a thicker alginate layer, which lead to an increase in the diffusion-bath length.

The release of the TEL encapsulated in the alginate beads was prolonged with an increased concentration of calcium chloride (X2). A higher percentage of cross-linker between 10% and 15% had a retarding effect on drug release. When a strong, rigid gel structure is formed around the matrix, diffusion is hindered and the dissolution medium cannot penetrate into the matrix at a high speed, resulting in a reduced release rate. A lower concentration of cross-linker (X2) formed a loose gel; consequently, the drug diffused easily from the matrix.

The drug release (Y3) was observed to be increased when the oil concentration (X3) was increased up to a moderate level, followed by a reduction in the drug release when the amount of oil was further increased. This may be attributed to the fact that the addition of oil (X3) may form an additional diffusion layer, resulting in slow drug release from the prepared beads [48]. The polynomial fitting model for in vitro drug release Q6h (Y3) is shown below.

$$\begin{array}{cc} \mathrm{Drug}\;\mathrm{release}\;Q6h\; & \left(\%\right)\\ & =69.0418+\left(-0.935248\right)\times {\;X}_{1}+\left(-13.8458\right)\times {\;X}_{2}+0.65901\times {\;X}_{3}\\ & +3.875\times {\;X}_{1}\times {\;X}_{2}+\left(-0.375\right)\times {\;X}_{1}\times {\;X}_{3}+2.125\times {\;X}_{2}\times {\;X}_{3}+\left(-6.33132\right)\times {\;X}_{1}^{2}\\ & +\left(-6.86165\right)\times {\;X}_{2}^{2}\;+\left(-7.2152\right)\times {\;X}_{3}^{2}\; \end{array}$$

##### ANOVA Analysis

After obtaining responses, the adjusted and predicted R2 was found using a central composite design, and ANOVA was used to figure out what the results mean. Table 3 shows the *p* values, precision, percentage of CV, adjusted R^2^, and predicted R^2^.

### 3.2. Optimization of the Formulation Factors Using the Central Composite Design

Using the central composite design, a numerical-optimization technique based on desirability was used to find the best formulation. The goals of the optimization study were to obtain the maximum entrapment efficiency and buoyancy and to get the drug to stay in the body for longer at Q6h. Table 4 shows that the best levels of the formulation factors for an optimized formulation based on the central composite design were 4.56% sodium alginate, 8.72% calcium chloride, and 7.68% oil with predicted values of 95.95% for entrapment efficiency, 10.89 h for in vitro buoyancy, and 66.00% for drug release Q6h with high desirability 0.978.

The optimized formulation was prepared using the emulsion-gelation technique, and the actual values of the responses were 98.84% for entrapment efficiency, 11.25 h for in vitro buoyancy, and 67.76% for drug release Q6h. it was found that the actual values were found to be close to the predicted values, which indicated the validity of the central composite design [52].

### 3.3. Study of the Effect of Oil on the Formulation of Gastroretentive Alginate Beads

The optimized formulation derived from the central composite design was formulated and compared to the same formulation without oil, and the effect of the oil on various parameters of the beads was studied.

#### 3.3.1. The Average Size of the Optimized Formulation (OP1 and OP2)

As represented in Table 5, the particle sizes of the beads were 1.22 ± 0.24 and 0.933 ± 0.06 mm for OP1 and OP2, respectively; the sizes of the gel beads increased with the oil entrapment. The increase in bead size may be attributed to the homogenous dispersion oil phase in the alginate solution. These oil droplets united to form larger droplets, increasing the sizes of the beads.

#### 3.3.2. The Entrapment Efficiency Percentage of the Optimized Formulation (OP1 and OP2)

The results in Table 6 show that the drug entrapment was higher for the oil-entrapped beads (OP1) compared with the beads without oil (OP2). The entrapment efficiency increased with the increase in oil concentration; a valuable explanation for this would be the hydrophobic nature of TEL. The drug partitions in the oil phase were due to its high lipophilic nature. In the formulation without oil, the drug partitions in the alginate matrix depended upon the concentration of the polymer [53].

#### 3.3.3. In Vitro Buoyancy of Optimized Formulation (OP1 and OP2)

The incorporation of oil imparts a pivotal role in the buoyancy of the beads. The results demonstrated that the oil-entrapped beads (OP1) exhibited a longer floating time of more than 11 h with a negligible lag time compared to the beads formed without oil loading, which floated for 6 h with an increased lag time (Table 7).

The emulsification of the sesame oil in the alginate solution and the rapid gelation within the alginate gel matrices resulted in the formation of tiny oil pockets either deep within the matrices or on the surfaces of the beads. These oily pockets offered buoyancy to the beads, allowing them to float for longer periods and providing a safety margin over premature sedimentation [54].

#### 3.3.4. The Swelling Ratio of the Optimized Formulation (OP1 and OP2)

As shown in Table 8, the swelling study of the optimized formulation exhibited a convincing swelling behavior for 6 h. The hydration of the alginate hydrophilic groups caused the dry beads to swell due to differences in the osmotic pressure of the fluid inside and outside the beads [55]. The bead swelling was the most significant behavior that influenced the drug-release pattern of the polymeric materials. The concentration of oil had a minimal effect on the swelling of the beads; as the concentration of oil increased, the swelling ratio of the beads decreased. These results may have been due to the presence of oil acting as a barrier for water absorption [48].

#### 3.3.5. In Vitro Release Study of the Optimized Formulation (OP1 and OP2)

The results of the in vitro drug release study demonstrated that the oil-entrapped beads had a prolonged drug release over 12 h in the simulated gastric fluid and that the cumulative release was 92.68 ± 3.09%. The entrapment of oil in the system enhanced the buoyancy of the beads, aiding in the floatation and increasing the amount of drug released. The beads without oil inclusion showed floating behavior for 7 h and the cumulative drug release was 91.88 ± 1.69%. The apparent area under the dissolution curve (AUC, or the integration of the drug release over the investigated periods of time) of the pure TEL, OP1, and OP2 was calculated as 184.32, 686.51, and 436.43 respectively.

TEL is a weakly basic drug exhibiting pH-dependent solubility. The drug is soluble at an extreme pH of 1.2 and precipitates on higher pH values. The extent of ionization is high in simulated gastric fluid; therefore, small soluble molecules tend to diffuse easily from the gel matrix. This explains the greater drug release seen with the oil-entrapped beads; in addition, the incorporation of oil into the beads increased the duration of the exposure of the beads to the acidic medium, thereby improving the solubility of the drug and its absorption [56].

**Figure 8:**
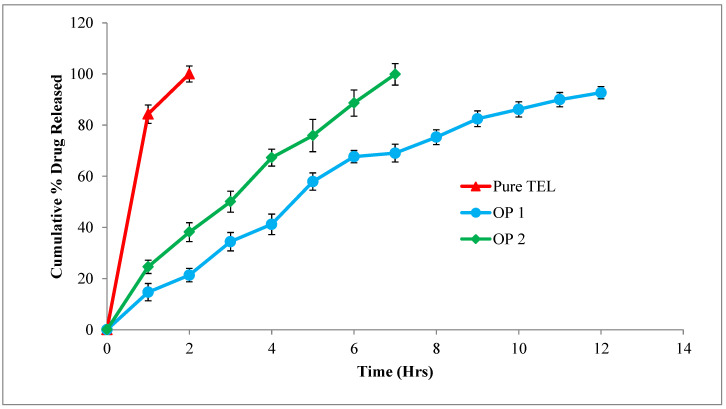
In vitro drug-release study of optimized formulation (OP1 and OP2) in comparison with pure TEL using simulated gastric fluid (pH 1.2) as dissolution medium.

According to the release profile, the oil-entrapped beads (OP1) had a greater sustaining activity than the conventional alginate beads (OP2) and pure TEL, as shown in Figure 8. The drug release from the oil-entrapped beads (OP1) was sustained sufficiently for a longer period of time in the simulated gastric juice when compared to the conventional (OP2) beads [57]. An additional property of buoyancy was observed in the oil-entrapped beads; this was due to the incorporation of oil, which has less density than water. The lower the density of the oil, the lower the amount of oil required to give it a buoyant nature [54]. Thus, it is evident that the oil-entrapped beads exhibited satisfactory gastro-retention and improved the solubility and drug release within the gastric region [58]. 

##### The In Vitro Release Kinetics of the Optimized Formulation

**Figure 9:**
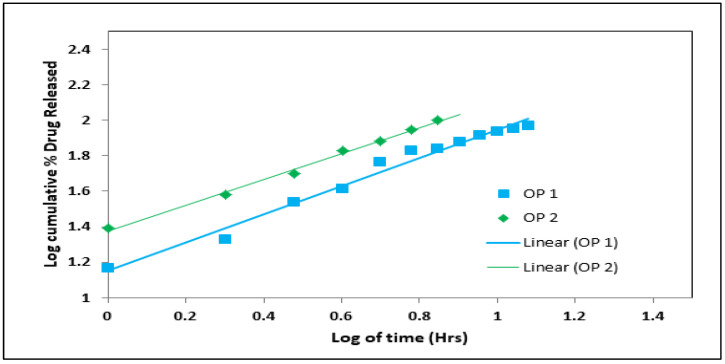
Korsemeyer–Peppas plot of the optimized formulation (OP1 and OP2).

Several kinetic models were tested using the values from the in vitro dissolution of the optimized formulation (OP1 and OP2). Different kinetic equations were used to fit the data from the in vitro release to figure out how the drug was released and how fast it was released. Table 9 and Figure 9 show the results.

The in vitro release of the best formulation showed that the TEL-loaded alginate beads could release the most drug from the calcium alginate matrix in the acidic medium (pH 1.2). The release of the drug was controlled by how TEL dissolve in the dissolution medium and how the drug diffuse through the polymer matrix.

Table 9 shows how different kinetic release models, such as the zero-order, first-order, Higuchi, Korsemeyer–Peppas, and Hixson–Crowell models, were used with the data from the in vitro release tests to figure out how the drug was released from the floating gel beads. All the kinetics equations and graphs are presented in Supplementary File. Regression coefficients and slopes (rates) were compared to see what effect they had on drug release.

The drug release from the beads followed the Higuchi model, not the zero-order or first-order equations, as shown by the higher correlation coefficient (r^2^ = 0.9836). Based on these results, it seems that diffusion control was used to control how much drug was released from the oil-entrapped beads. Diffusion, swelling, and erosion are the three most important rate-controlling mechanisms used in controlled-release formulations [59]. The release of drugs from the polymeric system occurs mostly by diffusion, which can be most properly depicted using Fickian diffusion [59]. However, with regard to formulations with swelling polymers, processes other than diffusion, such as the relaxing (erosion) of polymer chains and the imbibing of water, which causes polymers to expand, play a crucial part in determining the drug-release mechanisms. Swelling causes a significant volume expansion, which causes diffusion boundaries to move, making it more difficult to satisfy Fick’s second equation of diffusion [60]. Therefore, the Korsmeyers–Peppas model gave the equation that was used to do more work with the release data. In the Korsmeyer–Peppas model, the value of the release exponential n got close to n = 0.836, which is the value for a super case II transport mechanism. This means that swelling, erosion, and diffusion all work together. The drug got out of the beads because of the controlled polymer dissolution, polymeric chain expansion, or polymer relaxation or swelling. The results show that polymer swelling and relaxation, followed by diffusion, were the main factors that controlled drug release. Similar results were obtained by Manjanna et al. (2013) [61].

#### 3.3.6. The Surface Morphology of the Optimized Formulation Using SEM 

**Figure 10:**
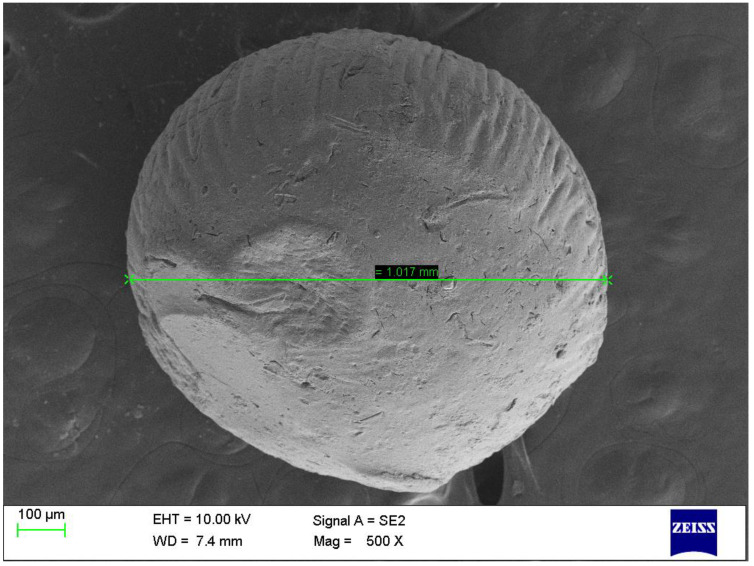
SEM image of the optimized formulation.

As shown in Figure 10, the SEM image of the optimized formulation of TEL-loaded oil-entrapped floating alginate beads have an almost spherical shape with rough and porous exterior surfaces, which assisted in the controlled release of the drug from the floating beads with good buoyancy. The results were in agreement with the findings obtained by Treesinchai et al. in 2019 [54].

The cracks and fissures on the outside could have been caused by the movement of water molecules during drying and the subsequent shrinking of the polymeric gel [62]. It can be seen that the polymers were successful in their cross-linking with Ca^2+^, which led to the uniform creation of the spheres. The fact that there were no crystals of the drug visible on the surfaces of these beads suggested that the drug was present in the alginate matrix in a finely distributed condition. These results in good agreement with the findings of Ismail et al. (2019) [63].

#### 3.3.7. The Compatibility between Drug and Ingredients in the Optimized Formulation by FTIR Spectroscopy

**Figure 11:**
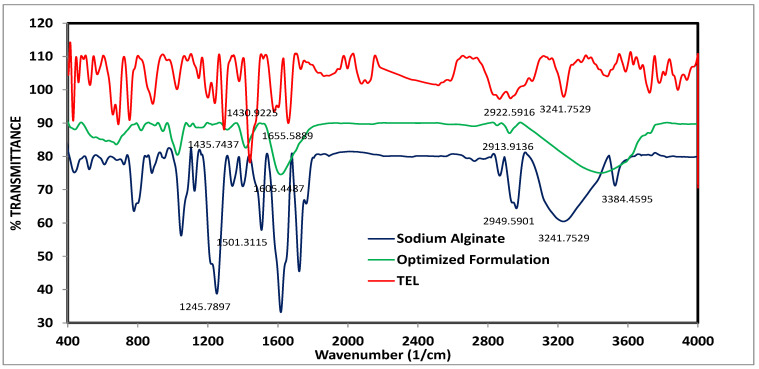
The IR spectra of TEL, sodium alginate, and the optimized formulation.

The IR spectroscopy was used to study the possible interactions between TEL and the sodium alginate in the formulation. As shown in Figure 11, the FT–IR spectrum of the pure drug that exhibited a characteristic peak at 3241.75 cm^−1^ was attributed to the O-H stretching of –COOH; N-H stretching was observed at 2922.59 cm^−1^ and the aromatic C-H stretching vibrations were observed at 1655.58 cm^−1^. Furthermore, the bands at 1430.92 cm^−1^ were assigned to the C-N stretching of the imidazole ring of TEL. The results were in agreement with the findings obtained by Sharma et al. (2019) [64]. The spectrum of sodium alginate had predominant peaks at 3410 cm^−1^ for the O-H stretching and the 3384.45 cm^−1^ attributed to the N-H stretching; the peaks at 1501.31 and 1245.78 cm^−1^ were attributed to the C-C and C-H groups. The results were in agreement with the findings obtained by Azad et al. (2021) [62]. The IR spectrum of the optimized formulation showed all characteristic peaks of TEL, which indicted the compatibility between TEL and other ingredients as well as the absence of any interaction.

### 3.4. In Vivo Pharmacokinetics Evaluation Study

**Figure 12:**
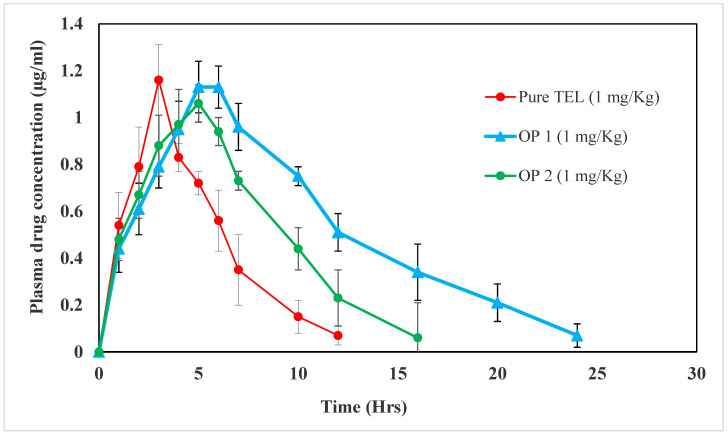
Plasma-concentration-time curve after oral administration of pure TEL, OP1, and OP2.

Improvements in the oral bioavailability of the drug were the primary goal of the TEL beads prepared in the present study, in which a biodegradable polymer was used. Indeed, the assessment of drug bioavailability is an essential parameter to be considered when preparing new dosage forms. Table 10 and Figure 12, demonstrate the plasma levels of the pure TEL drug and TEL beads (OP1 and OP2) after a single dose of 1 mg/kg. The C_max_ was observed for the TEL beads at 1.13 ± 0.08 and 1.06 ± 0.15 µg/mL for OP1 and OP2, while for the pure TEL it was 1.16 ± 0.15 µg/mL. The time required to reach the maximum concentration was the T_max_. According to the results, it was found that the T_max_ was 3, 5, and 5 h for the pure TEL, OP1, and OP2, respectively. These results may be attributed to the slow diffusion of TEL from the prepared beads [65]. Additionally, the K_el_ of the TEL beads (OP1 and OP2) were 0.0585 ± 0.005 and 0.093 ± 0.007 h^−1^, which is markedly lower than that of the pure drug (0.144 ± 0.02 h^−1^). Furthermore, the improved area under the curve (AUC_0–∞_) (12.495 ± 1.56 µg·h/mL) was observed in the TEL-loaded beads with oil, while for the beads without oil, it was 8.25 ± 10.03 µg·h/mL. It was found that the AUC for TEL beads OP1 or OP2 was higher than for the pure drug (5.615 ± 0.928 µg·h/mL). The relative bioavailability percentages of the OP1 and OP2 were 222.52 ± 20.74% and 146.93 ± 18.35%, respectively. This observed enhancement in bioavailability can be attributed to the prolonged release and enhanced absorption rate of the drug. The mean residence times (MRTs) of the OP1 and OP2 were 9.02 ± 1.38 and 6.38 ± 0.85 h; these were higher than those of the pure TEL, which was 1.86 ± 0.26 h. These results may be attributed to the floating of the prepared alginate beads compared to the pure drug [43,65]. The practical pharmaceutical implication of the TEL beads with oil showed a more favourable bioavailability. This could be attributed to the formation of a supersaturated solution, which can significantly enhance the absorption and bioavailability [51].

### 3.5. Anti-Hypertensive Efficiency of Gastro-Retentive Beads

**Figure 13:**
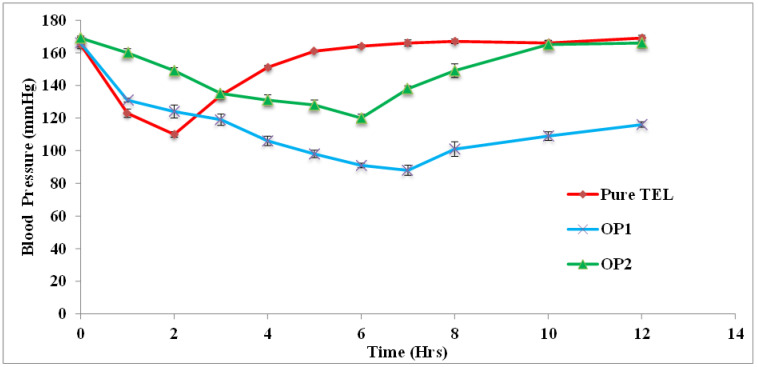
Antihypertensive effect of optimized TEL-loaded beads (OP1 and OP2) in hypertension-induced rats.

The anti-hypertensive activity of the TEL-loaded gastro-retentive beads prepared with and without oil entrapment was studied in the rat animal model. The blood pressure was monitored and measured at different time intervals following the treatments. The results of the pharmacodynamics study groups are depicted in Table 11 and Figure 13.

The results displayed the anti-hypertensive effect of the beads after inducing BP in the rats using MPA. The administration of MPA produced a significant rise in BP in all of the four groups; the positive control was found to be statistically significant when compared with the control group, indicating the validity of the model. The oral administration of the pure TEL significantly controlled the BP during the initial hour (*p* < 0.05). The pure TEL exerted its maximum effect up to 2 h, after which the BP rose gradually. 

The administration of the beads formulation without oil (OP2) displayed a gradual decrease in BP up to 6 h; the maximum effect of the formulation was observed at the 6th h with a significant *p* value (*p* < 0.05). After the peak effect at the 6th h, the BP rose to the initial value. 

After the administration of oil-entrapped beads (OP1), the BP was significantly reduced in the initial hour and the effect was continuous over a period of 12 h. The maximum effect was observed at the 7th h; the result was significant with a value of *p* < 0.05. According to these results, the administration of pure TEL controlled the BP quickly and drastically, but its effect wore off after 4 h. However, the bead formulation without oil (OP2) controlled the BP gradually and only for a period of 6 h. The oil-entrapped beads (OP1) resulted in a continued drug release over 12 h and the beads were able to control hypertension throughout the period, showing a sustained anti-hypertensive effect. These results may have been due to the slow diffusion of the drug, resulting in prolonged effects for 12 h [66].

From the study, it was evident that the bead formulation overcame the limitations of conventional formulations of TEL, such as low solubility and lower bioavailability. The oil-entrapped-beads formulation (OP1) exerted a significant effect over a longer period by continuously releasing the drug for 12 h.

## 4. Conclusions

The current study aimed to develop and optimize TEL-loaded oil-entrapped floating alginate beads in order to prolong the residence time of drugs in the gastric region, thereby improving their solubility and bioavailability. Because of the gastro-retentive qualities of alginate beads, it is possible to control the systemic absorption of TEL, as well as improve and prolong it. The conclusions that can be drawn from the findings of the in vivo trials are that the anti-hypertensive action of this formulation is maintained and that it considerably increases TEL’s therapeutic efficacy.

## Figures and Tables

**Table 1:** The formulation factors and responses based on the central composite design for TEL-loaded oil-entrapped floating alginate beads.

Formulation Factors (Independent Variables)	Low (−1)	Med (0)	High (+1)	−Alpha (−1.68179)	+Alpha (+1.68179)
X1: Sodium alginate concentration (%)	2	4	6	0.636414	7.36359
X2: Cross-linker concentration (%)	5	10	15	1.59104	18.409
X3: Sesame oil concentration (%)	4	6	8	2.63641	9.36359
Responses (dependent variables)	Goals				
Y1: Entrapment efficiency (%)	Maximize				
Y2: In vitro buoyancy (h)	Maximize				
Y3: Drug release Q6h (%)	Prolong				

**Table 2:** The composition of the designed formulations and measured responses of the prepared TEL-loaded oil-entrapped floating alginate beads according to central composite design.

Std	Run	Independent Variables			Dependent Variables		
		X1	X2	X3	Y1	Y2	Y3
13	1	0	0	−1.68179	59	2	49
16	2	0	0	0	90	9	69
7	3	−1	1	1	66	8	35
3	4	−1	1	−1	41	2.5	28
1	5	−1	−1	−1	50	4	66
17	6	0	0	0	90	9	69
8	7	1	1	1	92	10.6	42
11	8	0	−1.68179	0	56	6.2	75
9	9	−1.68179	0	0	51	3.3	55
6	10	1	−1	1	83	10	56
4	11	1	1	−1	77	2.6	36
14	12	0	0	1.68179	94	11.5	49
10	13	1.68179	0	0	90	7	48
12	14	0	1.68179	0	63	7.5	25
2	15	1	−1	−1	63	4.5	59
5	16	−1	−1	1	68	7	65
15	17	0	0	0	90	9	69

**Table 3:** ANOVA analysis for optimization of responses.

Responses	Adjusted R^2^	Predicted R^2^	Model *p* Value	Adequate Precision	% CV
Entrapment efficiency (Y1)	0.9901	0.9613	<0.0001	39.06	2.40
In vitro buoyancy (Y2)	0.9750	0.9167	<0.0002	25.34	7.20
Drug release Q6h (Y3)	0.9830	0.9434	<0.0001	30.15	3.82

**Table 4:** The composition and observed and predicted values of the optimized formulation based on desirability using the central composite design.

Factor	Alginate (X1)	CaCl_2_ (X2)	Sesame Oil (X3)	Desirability
Optimized formulation (OP1)	4.56%	8.72%	7.68%	0.978
Point Prediction	Y1	Y2	Y3	
Predicted	95.95	10.89	66.0	
Observed	98.84	11.25	67.76	

**Table 5:** Mean particle size of optimized formulation (OP1 and OP2).

The Optimized Formulation	Mean Particle Size (mm)
Optimized beads with oil (OP1)	1.22 ± 0.24
Optimized beads without oil (OP2)	0.933 ± 0.06

**Table 6:** The entrapment efficiency of the optimized formulation (OP1 and OP2).

The Optimized Formulation	Entrapment Efficiency (%)
Optimized beads with oil (OP1)	98.83 ± 1.23%
Optimized beads without oil (OP2)	90.35 ± 3.56%

**Table 7:** In vitro floating of optimized formulation (OP1 and OP2).

The Optimized Formulation	Floating Lag Time (s)	Floating Time (h)
Optimized beads with oil (OP1)	5.1 ± 2	11 ± 2.45
Optimized beads without oil (OP2)	12 ± 1.5	6.7 ± 1.7

**Table 8:** Swelling ratio of optimized formulation (OP1 and OP2).

The Optimized Formulation	Swelling Ratio					
	Time (h)					
	1	2	3	4	5	6
Optimized beads with oil (OP1)	1.62	1.58	1.53	1.49	1.47	1.41
Optimized beads without oil (OP2)	1.76	1.68	1.64	1.59	1.55	1.52

**Table 9:** In vitro release profile of optimized formulation (OP1 and OP2) in various models.

Kinetic Models	K		R^2^		n	
	OP1	OP2	OP1	OP2	OP1	OP2
Zero-order	7.334	13.68	0.9524	0.9852		
First-order	0.0939	0.0939	0.9799	0.6402		
Higuchi kinetics	34.416	38.165	0.9836	0.9653		
Korsemeyer–Peppas	1.1548	1.375	0.9762	0.9816	0.836	0.7297
Hixson–Crowell	0.269	0.5145	0.6982	0.7091		

**Table 10:** The pharmacokinetic parameters of TEL, OP1, and OP2.

Pharmacokinetic Parameters	Pure TEL	OP1	OP2
C_max_ (μg/mL)	1.16 ± 0.15	1.13 ± 0.08	1.06 ± 0.15
T_max_ (h)	3	5	5
AUC_0–∞_ (µg·h/mL)	5.615 ± 0.928	12.495 ± 1.56 *	8.25 ± 1.03
K_el_ (h^−1^)	0.114 ± 0.02	0.0585 ± 0.005 *	0.093 ± 0.007
Relative bioavailability (%)	-	222.52 ± 20.74 *	146.93 ± 18.35
MRT (h)	1.86 ± 0.26	9.02 ± 1.38 *	6.38 ± 0.85

All values are expressed as mean ± SD (n = 3); * significant OP1 when compared with OP2 formulation (*p* < 0.05).

**Table 11:** Antihypertensive effect of optimized TEL-loaded beads (OP1 and OP2) in hypertension-induced rats.

Mean BP (mmHg)	Group I	Group II	Group III	Group IV	Group V
	Control	Positive Control	Standard	OP2	OP1
Initial (h)	125 ± 1.5	167 ± 1.2	165 ± 3.4	169 ± 3.2	166 ± 1.5
1	124 ± 2.6	163 ± 2.6 *	123 ± 1.2 **	160 ± 1.5 ***	131 ± 3.1 ****
2	125 ± 1.7	162 ± 1.9 *	110 ± 4.0 **	149 ± 2.6 ***	124 ± 3.1 ****
3	124 ± 2.4	165 ± 1.3 *	134 ± 3.5 **	135 ± 2.5 ***	119 ± 4.1 ****
4	125 ± 1.2	164 ± 3.2 *	151 ± 2.8 **	131 ± 4.1 ***	106 ± 2.3 ****
5	123 ± 0.6	163 ± 3.1 *	161 ± 2.3	128 ± 5.1 ***	98 ± 3.2 ****
6	125 ± 1.0	165 ± 2.4 *	164 ± 1.4	120 ± 6.3 ***	91 ± 1.9 ****
7	124 ± 1.9	166 ± 1.8 *	166 ± 3.1	138 ± 3.9	88 ± 3.2 ****
8	126 ± 1.4	167 ± 4.2 *	167 ± 4.5	149 ± 1.3	101 ± 6.3 ****
10	122 ± 1.7	167 ± 2.3 *	166 ± 2.6	165 ± 4.1	109 ± 6.4 ****
12	125 ± 1.7	168 ± 3.1 *	169 ± 1.6	166 ± 1.9	116 ± 7.2 ****

BP—blood pressure. All values are expressed as mean ± SD (n = 3); * significant when compared with control (*p* < 0.05); ** significant when compared with positive control (*p* < 0.05); *** significant when compared with positive control (*p* < 0.05); **** significant when compared with positive control (*p* < 0.05).

## Data Availability

Data are available from the corresponding author upon request.

## Supplementary Materials

The following supporting information can be downloaded at: https://www.mdpi.com/article/10.3390/pharmaceutics15020709/s1, File S1: In vitro drug release kinetics analysis.
