# Supplementary material for: Optimization and In Vitro Characterization of Telmisartan Loaded Sodium Alginate Beads and Its In Vivo Efficacy Investigation in Hypertensive Induced Animal Model
Source: Pharmaceutics. 2023 Feb 20;15(2):709. doi: 10.3390/pharmaceutics15020709 (PMC9959044; doi:10.3390/pharmaceutics15020709)
Supplement: Supplementary file 1 [file pharmaceutics-15-00709-s001.zip › pharmaceutics-2132945-supplementary.pdf]

SUPPLEMENTARY DATA

Zero order kinetics

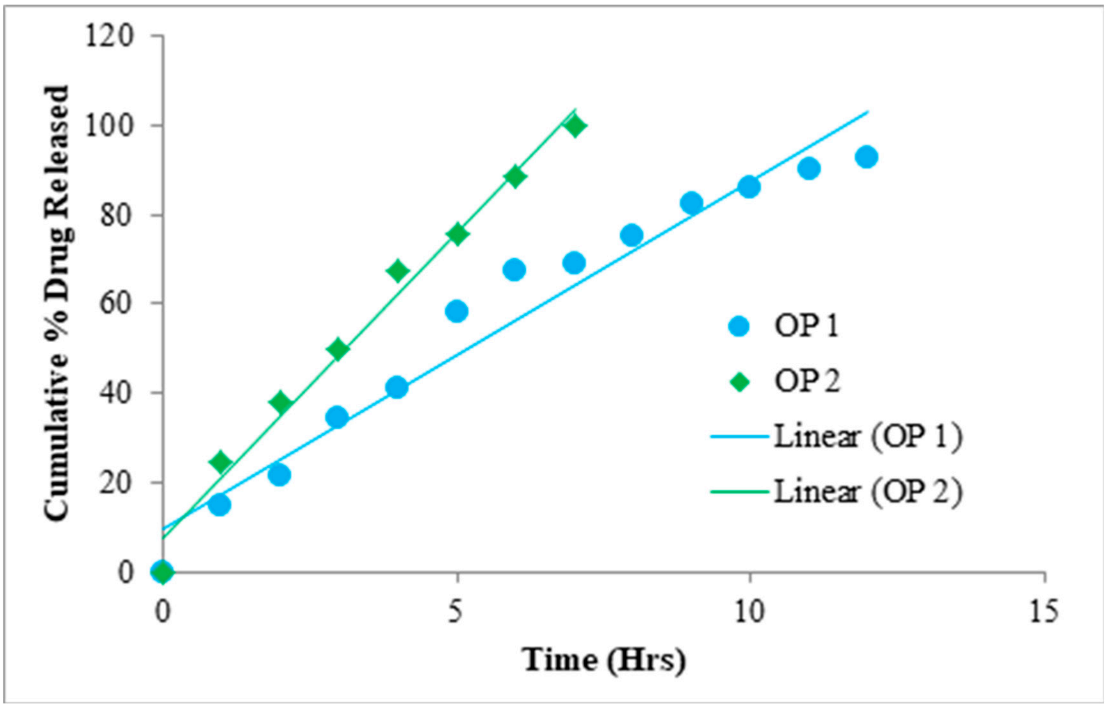

First order kinetics

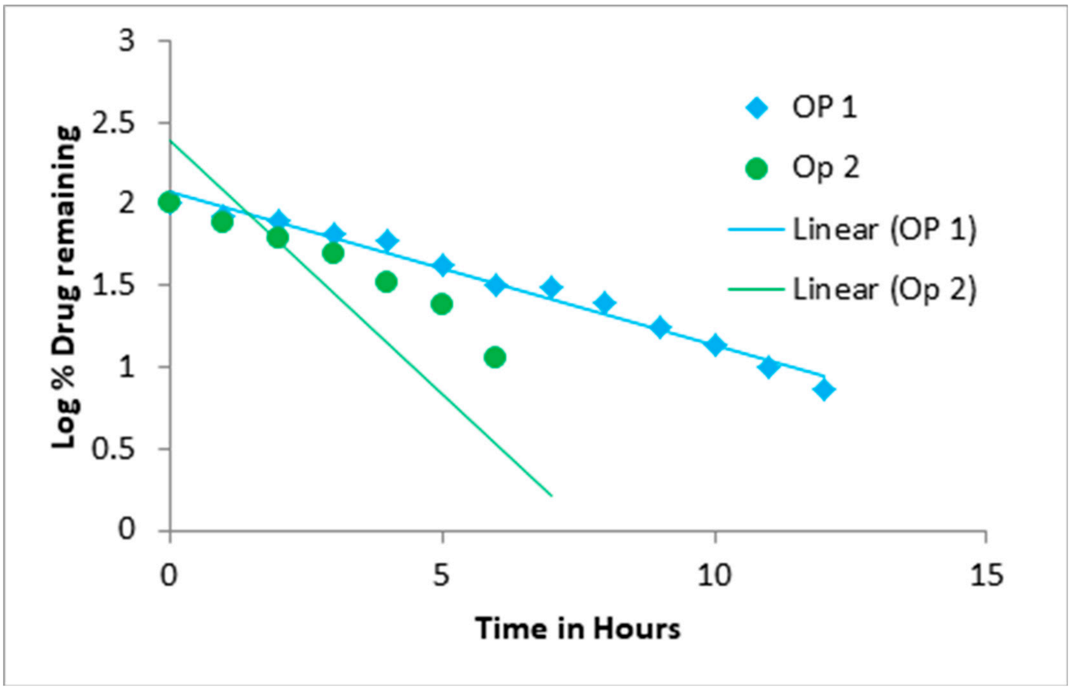

Higuchi kinetics

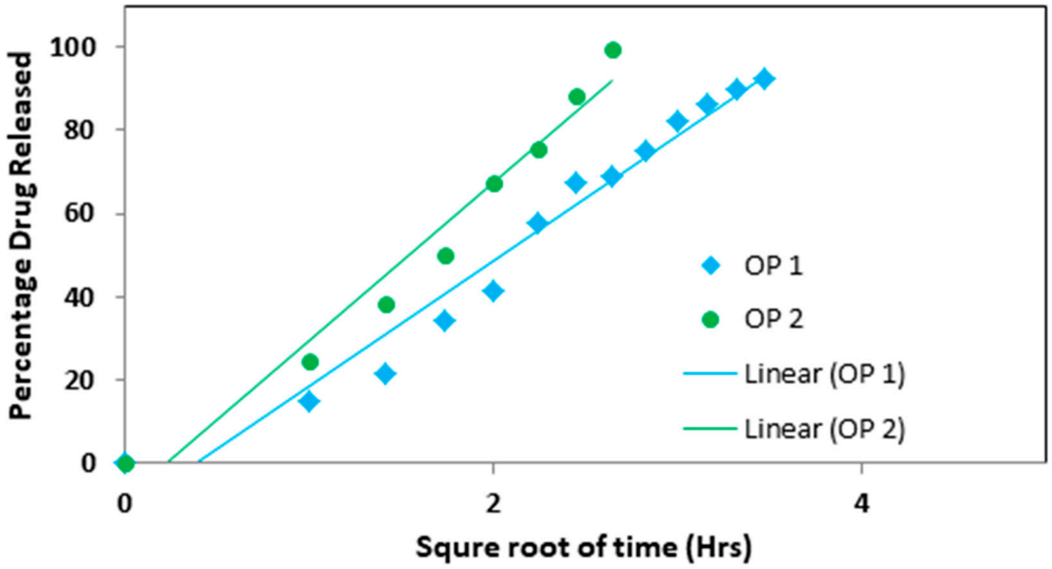

Korsmeyer-Peppas kinetics

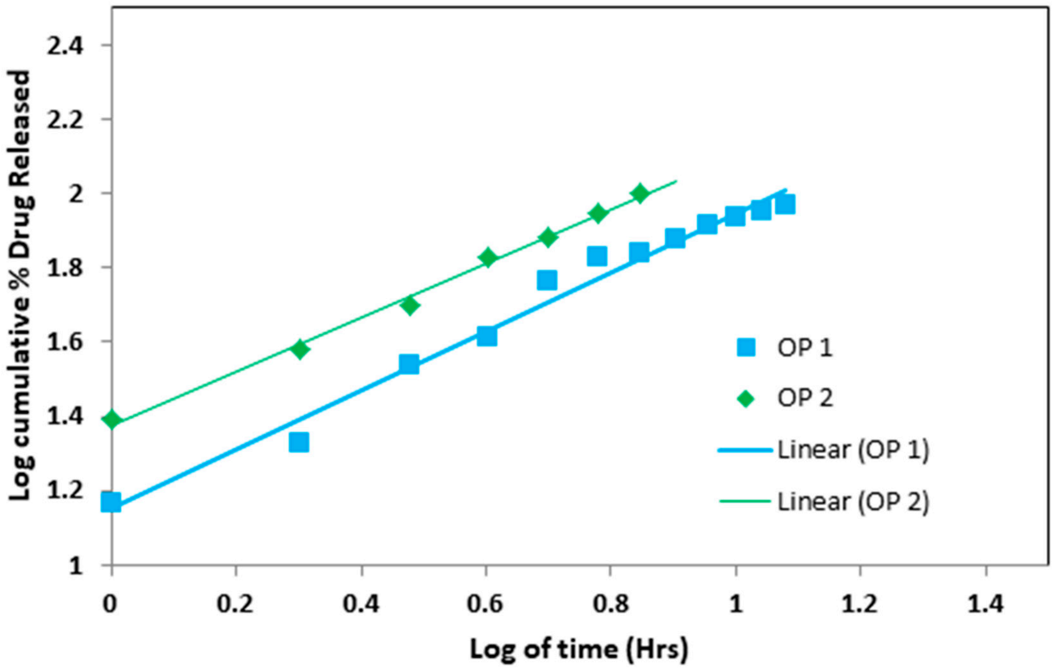

HIXSON-CROWELL Kinetics

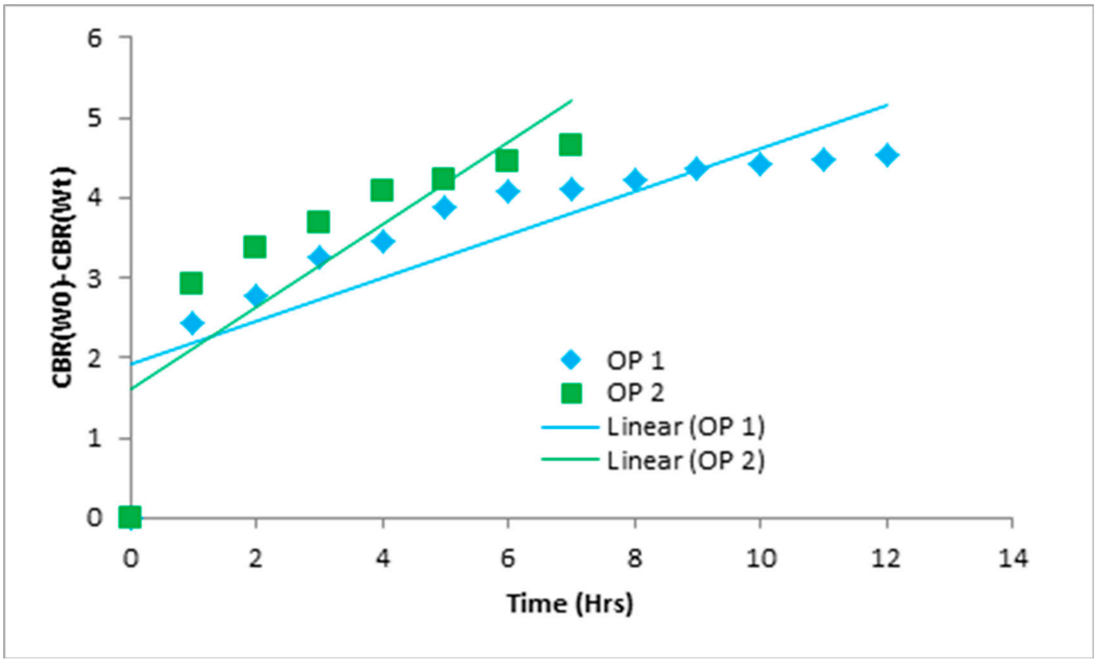

IN VITRO RELEASE KINETICS EQUATION

ZERO ORDER KINETICS

Zero order kinetics can be expressed by the equation,

$$Q_t = Q_0 + K_0 t$$

Where, Qt is the amount of drug released at time t,

K0 is the apparent dissolution rate constant or zero order release constant and

Q0 is the initial concentration of drug in the solution resulting from a burst effect; in this case the drug release runs at a constant rate. It describes the systems where the drug release rate is autonomous of its concentration of the dissolved substance. A graph is plotted between the time taken on x-axis and the cumulative % of drug release on y-axis and it gives a straight line.

FIRST ORDER KINETICS:

First order kinetics can be expressed by the equation,

$$dc/dt = -Kc$$

Where, K is first rate constant expressed in units of time^{-1}.

Equation can be expressed as:

$$\text{Log } C = \log C_0 - Kt/2.303$$

Where, C_0 is the initial concentration of drug,

K is the first order rate constant and

t is the time.

The data obtained are plotted as log cumulative percentage of drug remaining vs time, which would yield a straight line with a slope of – Kt/2.303.

HIGUCHI KINETICS

Higuchi kinetics can be expressed by the following equation,

$$Q = KH\ t^{1/2}$$

Where, Q is the cumulative amount of drug release at time t

KH is Higuchi constant

t is time in hours

The Higuchi equation suggests that drug release by diffusion. A graph is plotted between the square root of time taken on x-axis and the cumulative percentage of drug release on y-axis and it gives a straight line.

KORSEMEYER -PEPPAS EQUATION:

Korsemeyer -peppas equation can be expressed as,

$$\mathbf{F = (M_t/M) = K_m t^n}$$

Where, F is fraction of drug released at time t

Mt is amount of drug release at time t

M is total amount of drug in dosage form

Km is kinetic constant

n is diffusion or release exponent

t is time in hours

n is estimated from linear regression of log (Mt/M) vs log t.

If n= 0.45 it indicates fickian diffusion.

$0.45<n<0.89$ it indicates anomalous diffusion or non-fickian diffusion.

Anomalous diffusion or non- fiction diffusion refers to a combination of both diffusion and erosion controlled rate rules. A graph was plotted between the log time release on x-axis and the log cumulative percentage of drug release on y-axis and it gives a straight line [57].

HIXSON-CROWELL EQUATION:

Hixson-crowell equation can be expressed as,

$$W_0^{1/3} - W_t^{1/3} = \kappa\ t$$

Where, W_0 is the initial amount of drug in the pharmaceutical dosage form,

W_t is the remaining amount of drug in the pharmaceutical dosage form at time t and

K (kappa) is a constant incorporating the surface-volume relation.

The equation describes the release from systems where there is a change in surface area and diameter of particles. To study the release kinetics, data obtained from in vitro drug release studies were plotted as cube root of drug percentage remaining in matrix versus time and it gives a straight line.
